# Ectopic Molar with Maxillary Sinus Drainage Obstruction and Oroantral Fistula

**Published:** 2013-06

**Authors:** Shahin Abdollahifakhim, Mehrnoush Mousaviagdas

**Affiliations:** 1 *Departments of Otorhinolaryngolog, Tabriz University of Medical Sciences, Tabriz, Iran.*

**Keywords:** Ectopic tooth, Maxillary sinus ostia, Oroantral fistulae

## Abstract

**Introduction:**

Ectopic tooth eruption may result owing to one of 3 processes: developmentalDisturbance, iatrogenic activity, or pathologic process, such as a tumor or a cyst. In rare cases, occlusion of the sinus ostia may predispose a patient to develop a maxillary sinus mucocele. When the maxillary sinus is invaded, symptoms usually occur late in the process.

**Case Report:**

A 17 years old boy referred to department of Otolaryngology, Head and Neck Surgery of university of medical sciences, Tabriz_Iran in 2010 with chronic recurrent mucoprulent discharge from retromollar trigone , posterior to right superior alveolar ridge. CT scan revealed a dense mass resembling tooth, obstructing sinus ostium with homogenous opacity with ring enhancement, occupying whole sinus and expanding all walls. A Caldwell Luke approach in combination with endoscopy was selected.

**Conclusion:**

In the present patient, removal of ectopic tooth resolved the symptoms completely, the fistula obstructed and discharges discontinued. An ectopic tooth is a rare entity obstructing sinus ostium. The etiology of ectopic eruption has not yet been completely clarified, but many theories have been suggested,including trauma, infection, developmental anomalies and pathologic conditions, such as dentigerous cysts. In summary, although the ectopic teeth is rare but it would be assumed in presence of unilateral symptoms of sinonasal cavity. Therefore in peristant unilateral sinonasal symptoms we should complete examining of this site to rule out rare causes of these symptoms.

## Introduction

Ectopic tooth eruption may result from one of the 3 processes:developmental disturbance, iatrogenic activity, or pathologic process, such as a tumor or a cyst (1,2). Dentigerous cysts are caused by expansion of dental follicles resulting from accumulation of fluid between the tooth crown and epithelial components (3). The maxillary canine and mandibular third molar are most frequently involved (4-6). In such cases the tooth can migrate to ectopic areas such as maxillary sinus, nose and infraorbital area (7-9). In the maxilla, these teeth are often displaced into the maxillary sinus. The dentigerous cyst progresses slowly and may exist for several years without being noticed (3,5,6,10,11).

In rare cases, occlusion of the sinus ostia may predispose a patient to develop a maxillary sinus mucocele (12). When the maxillary sinus is invaded, symptoms usually occur late in the process (11).The sequelae of these cysts and ectopic teeth vary, from obstruction of the sinus to blindness (13). 

Ectopic teeth obstructing sinus ostium is a rare condition. It causes morbidities sometimes differentiated with the most common causes of sinus ostium obstruction like foreign bodies, sinonasal tumors of any origin, sinonasal polyposis and etc. Trauma, infection, genetic, teeth crowding, dense alveolar bone, iatrogenic causes like dentistry and developmental disorders are some causes of ectopic teeth in sinonasal cavity (14,15).

Here we report a patient who was referred because of chronic mucoprulent discharge from retromollar trigone. This condition was due to dentigerous cyst caused by an ectopic tooth near the sinus ostium, that was compelling with its drainage.

## Case Report

A 17 years old boy referred to department of Otolaryngology, Head and Neck Surgery of Tabriz University of Medical Sciences in Iran in 2010 with chronic recurrent mucoprulent discharge from retromollar trigone, posterior to right and to superior alveolar ridge. The patient also had fullness sensation on his right cheek, halitosis, posterior nasal discharge, pain and swelling around a fistulous site where drainage occurred. Office examination revealed a 2 mm point with mucoprulent discharge in retromolar trigone. A 4 mm 0 degree rigid rod endoscope introduced to nasal cavity to rule out any obstructing mass in nasal cavity and middle meatus. 

Mucoprulent discharge from middle meatus was apparent in nasal endoscopy. Multiple courses of medical treatment with various generations of antibiotics had been used for 1years. Imaging like waters and panoramic X-ray revealed no definite diagnosis until he referred to our institute. He had no previous history of trauma, dentistry, infection and no evidence of developmental disorders.

**Fig 1:**
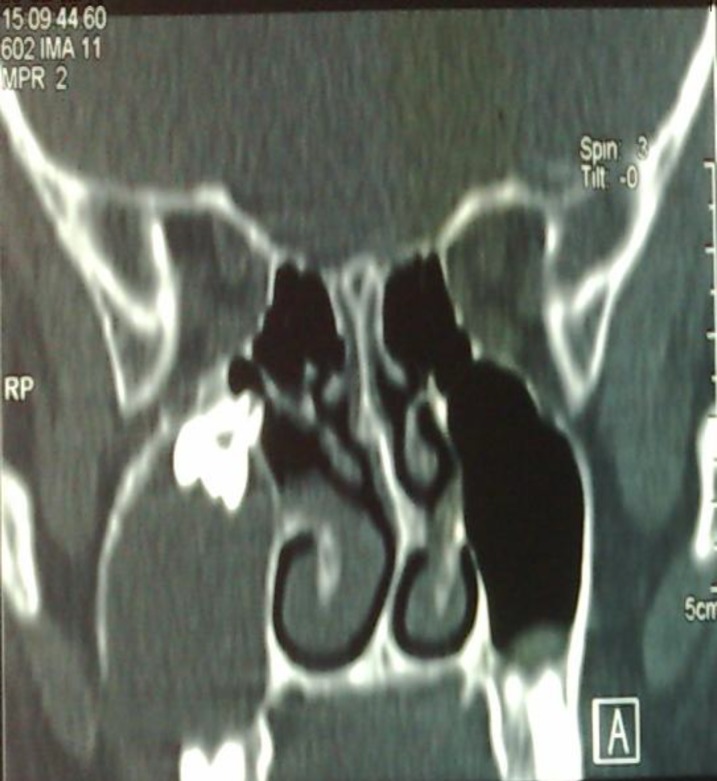
Ectopic tooth near ostium

Previous imaging was studied again and in panorex X-Ray an opaque mass was seen near the right maxillary sinus antrum, which was missed during previous visits. CT scan revealed a dense mass resembling tooth,near the accessory sinus ostium with near total opacification of maxillary sinus. Anterior and lateral walls had been eroded in lower cuts (Fig. 1).

**Fig2:**
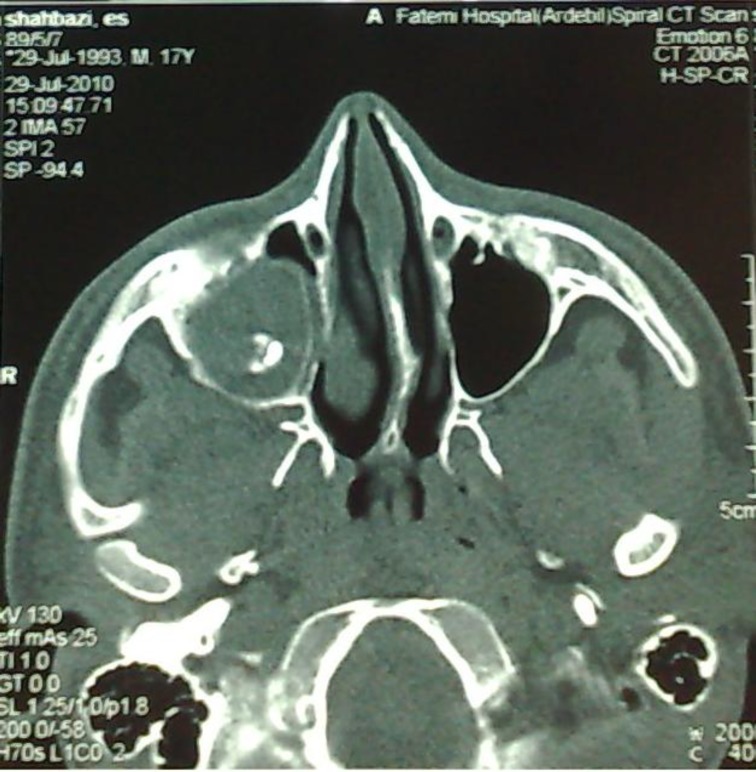
Maxillary Anterior wall Erosion

**Fig3:**
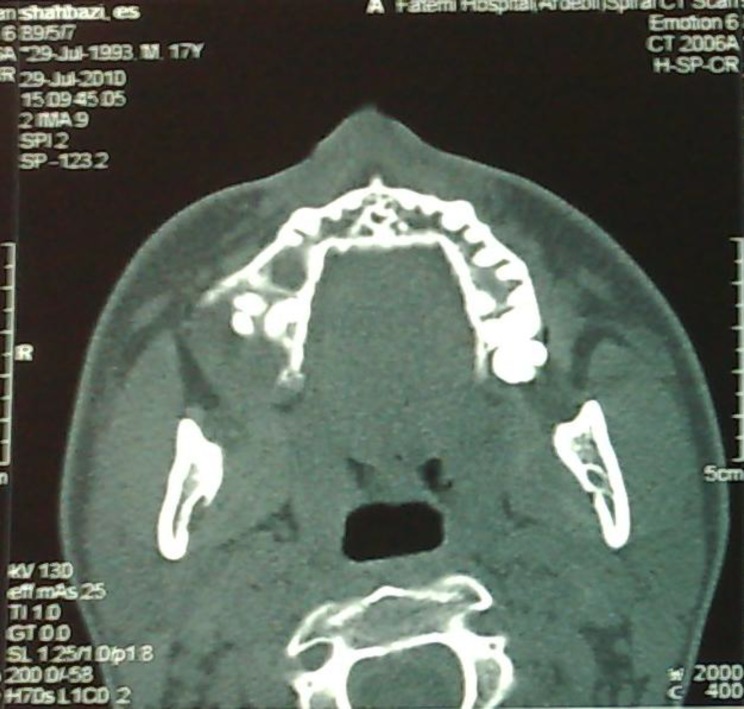
Lateral Maxillary wall Erosion

A Caldwell Luke approach in combination with endoscopy was selected. A sub labial incision made, preserving 2 mm of mucosa on gingival side. Dissection was made in sub periosteal plane, up to infra orbital bundle. Erosion was seen on anterior wall of maxillary sinus. The anterior wall was fenestrated with a 4 mm direct osteotome. A cystic mass in grayish color filled with mucoid secretions dissected from sinus walls, erosion in lateral and posterior wall was seen. The ectopic tooth has been placed near the sinus ostium, adjacent to floor of the orbit, compelling with sinus drainage. Using a 4 mm rod lens endoscope, the ectopic tooth extracted and uncinectomy and antrostomy was completed.

A fistulous tract was found in floor of sinus draining to retromolar trigone. It was sealed off with muscle & adipose tissue .The ectopic tooth had four roots and it was consistency lesser than normal teeth.

Because of negative history of tooth extraction and location of fistula in retromolar trigone, it could not be due to tooth eruption. Pathologic examination revealed dentigerous cyst and no evidence of dental origin mass lesions was found.

## Discussion

An ectopic tooth is a rare entity obstructing sinus ostium. The etiology of ectopic eruption has not yet been completely explained, but many theories have been suggested, including trauma, infection, developmental anomalies and pathologic conditions, such as dentigerous cysts (16). The etiology of ectopic teeth in the maxillary sinus in our patient is unknown. Ectopic molars and supernumerary teeth are usually discovered on routine radiographic examinations, because most of the cases are asymptomatic (17). The dentigerous cyst progresses slowly and may exist for several years without being noticed. When the maxillary sinus is invaded, symptoms usually occur late in the process (11). But when the cyst enlarge and teeth migrate, patient may experience significant morbidity and require intervention. An ectopic tooth in the maxillary sinus causes several common problems. The most frequent of which are facial pain (1,6,18), facial edema (1,4,5,10,13,18-21), rhinorrhea, headache and nasolacrimal obstruction (6,19,22,23). A large maxillary cyst involves the whole sinus and can transmit pressure to the walls of sinus; consequently, ophthalmologic and nasal symptoms may develop (19). Moreover, various findings are described: epiphora, superior orbital fissure syndrome, eruption into the orbit causing blindness (12) sinusitis or nasal obstruction, odontogenic cyst, an oroantral fistula, other missing teeth, and sepsis (12,14,17). Our patient was presented with oroantral fistula (to retromolar trigon) that is rare in literature review. 

Timur saieem reported a 45 years old man with 2-month history of haemoptysis. Morton Litwin in 2008 reported a 57 years old black woman with dentigerous cyst and an impacted tooth in orbital rim that presented with facial swelling (24,13). In 2011 Irfan Kaygusuz reported a mucocele associated with an ectopic tooth in the maxillary sinus (25), without fistula. The most common presentation of maxillary tooth in Buyukkurt’s report was facial and cheek swelling. Avitia et al reported a case of orbital proptosis resulting from a dentigerous cyst in the maxillary sinus associated with a displaced tooth (6,26).

Water view, panoramic radiography and plain skull radiography are simple and inexpensive methods that can be used in daily practice (27). CT scans provide superior bony detail, allowing for the visualization of the size and extent of the lesion with determination of orbital or nasal invasion or involvement (5). Therefore, CT may be more valuable than plain film radiographs, not only for definitive diagnosis, but also for evaluation of the associated pathology, exact localization of the ectopic tooth, and proper treatment planning (2,27). Patients with unilateral sign and symptoms of any sinonasal disorder should be investigated for sinonasal mass lesions like tumors primarily and then inflammatory or anatomical causes and sometimes, foreign bodies and exostoses.

The standard treatment for a dentigerous cyst is enucleation and extraction of the cyst-associated impacted or unerupted tooth (5,21,28,29). The surgical treatment in the maxillary sinus involves removal via a Caldwell–Luc procedure (14,30). Caldwell–Luc procedure was followed in this case, since the ectopic tooth was the cause of a maxillary sinus cyst. Jude et al reported a case of occlusion of sinus ostium by an ectopic molar that required an anterior antrostomy or Caldwell-Luc surgery (31). When the molar teeth is high in the medial wall of sinus (near to ostium) we can remove it endoscopically. Abdul Salam Hasbini in 2001and Alexandrakis et al in 2000 reported endoscopically removal of tooth (32,33).

Marsupialization is another advisable treatment to preserve the cyst-associated tooth and promote its eruption (34-36). Takagi and Koyama reported that marsupialization was useful for promoting eruption of an ectopic second premolar associated with dentigerous cysts in the maxillary sinus of a 6-year- old child (10). The major disadvantage of marsupialization is recurrence or persistence of the lesion (10,37,38). In the current case, removal of ectopic tooth resolved the symptoms completely. The fistula obstructed and the discharges discontinued.

## Conclusion

In summary, although the ectopic tooth is rare, it would be assumed in presence of unilateral symptoms of sinonasal cavity. Therefore in persistent unilateral sinonasal symptoms we should completely examine this site to rule out rare causes of these symptoms.
